# MicroRNAs as biomarkers for monitoring cardiovascular changes in Type II Diabetes Mellitus (T2DM) and exercise

**DOI:** 10.1007/s40200-022-01066-4

**Published:** 2022-07-07

**Authors:** Volga M Saini, Kaitlyn R. Liu, Aishwarya Suryakant Surve, Sanjeev Gupta, Ananya Gupta

**Affiliations:** 1 Discipline of Physiology, School of Medicine, NUI Galway, H91TK33 Galway, Ireland; 2 Discipline of Pathology, School of Medicine, NUI Galway, H91TK33 Galway, Ireland

**Keywords:** Type 2 diabetes mellitus, Diabetic heart disease, Circulating microRNA, Biomarker, Exercise.

## Abstract

**Introduction:**

MicroRNAs (miRNAs) have been shown to be altered in both CVD and T2DM and can have an application as diagnostic and prognostic biomarkers. miRNAs are released into circulation when the cardiomyocyte is subjected to injury and damage.

**Objectives:**

Measuring circulating miRNA levels in human plasma may be of great potential use for measuring the extent of damage to cardiomyocytes and response to exercise. This review is aimed to highlight the potential application of miRNAs as biomarkers of CVD progression in T2DM, and the impact of exercise on recovery.

**Methods:**

The review aims to examine whether the health improvements following exercise in T2DM patients are reflective of changes in expression of plasma miRNAs. For this purpose, studies were identified from the literature that have established a correlation between diabetes, disease progression and plasma miRNA levels. We also reviewed studies which looked at the effect of exercise on plasma miRNA levels.

**Results:**

The review identified miRNA signatures that are affected by T2DM and DHD and a subset of these miRNAs that are also affected by different types of exercise. This approach helped us to identify those miRNAs whose expression and function can be altered by regular bouts of exercise.

**Conclusions:**

miRNAs identified as part of this review can serve as tools to monitor the cardio-protective, anti-inflammatory and metabolic effects of exercise in people suffering from T2DM. Future research should focus on regulation of these miRNAs in T2DM and how they can be altered by appropriate exercise interventions.

**Supplementary Information:**

The online version contains supplementary material available at 10.1007/s40200-022-01066-4.

## Introduction

Epidemiological data has established that there is a strong association between type 2 diabetes mellitus (T2DM) and the onset of cardiovascular disease (CVD). Factors such as high blood sugar levels and high blood pressure can predispose diabetic patients to developing heart disease (termed diabetic heart disease or DHD). The risk of heart failure in diabetics remains constant and this could be due to the lack of potential biomarkers for monitoring disease progress and timely detection and diagnosis of ischemic changes. A better understanding of the modulators involved in DHD is required.

Type 2 diabetes mellitus (T2DM) is a chronic metabolic disorder whose global prevalence has progressively increased over the past years [1]. Diabetes Mellitus (DM) is a serious health concern for the public and is now considered a pandemic due to its financial burden on society and the significant health complications associated with those affected. For example, the International Diabetes Federation Diabetes Atlas estimated that by 2045, 693 million adults will be affected by DM [1]. T2DM is characterised by elevated glucose levels in the blood. This occurs as a result of deficient insulin secretion (from pancreatic β-cells) and failure of insulin-sensitive tissues to respond to insulin. This leads to the development of hyperglycaemia [2].

Chronic or late-stage diabetes is associated with a wide variety of complications. Among these, CVDs are the main cause of mortality and morbidity in diabetic patients. In fact, approximately 80% of diabetes-associated deaths are a result of heart disease [3].

Cardiovascular comorbidities play a key part in pathogenic pathways, with patients with diabetes having a 1.5–2-fold higher risk of high blood pressure, a 2–4-fold higher risk of coronary artery disease, and a 34% higher risk of atrial fibrillation [3]. Diabetes patients have a higher risk of myocardial infarction (77%) and ischemic stroke (68%) [3]. Diabetes-specific structural cardiac remodelling resulting in morphological and functional alterations, as well as activation of the renin–angiotensin–aldosterone system, excessive oxidative stress produced by hyperglycaemia, and insulin resistance, all contribute to a failing heart. According to the United Kingdom Prospective Diabetes Study, hyperglycaemia increases the risk of heart failure by 8% for every 1% increase in HbA1c [4]. These factors, when considered collectively, contribute to the 5-fold greater risk of heart failure reported in those with T2DM compared to those without [4]. Due to the poor health outcomes associated with diabetes, there is a need to identify new biomarkers to aid early diagnosis and monitor progression of these cardiovascular complications. This would greatly improve prognosis of chronic T2DM patients. Incidentally, studies such as those conducted by Lew et al. discovered that the cardiac dysfunction observed in diabetes correlates with altered expression of certain microRNAs (miRNAs/miRs) [4].

MicroRNAs (miRNAs) have been shown to be altered in both CVD and T2DM and can have an application as diagnostic and prognostic biomarkers. MiRNAs are a collection of conserved, single-stranded non-coding RNAs which are approximately 22 nucleotides long and are involved in regulating gene expression post-transcriptionally. They are involved in key physiological processes and their expression has found to be altered in disease states such as CVDs and DM [2, 5, 6]. Diabetes-induced molecular changes in the cardiac muscle activates a web of interrelated stress-signalling pathways that end in the activation of numerous transcription factors, co-regulators, including miRNAs. This altered gene expression contributes to the pathogenesis of the diabetic heart [2]. Several studies have shown that abnormal cardiac gene expression causes morphological and functional alterations in the heart, including hypertrophy, abnormal cardiac conduction, diminished contractility, and cardiomyocyte survival, as well as vascular homeostasis disruptions. This has led to the recent research focused on investigating the potential of miRNAs, specifically circulating miRNAs as diagnostic and prognostic biomarkers in diseases including CVDs [7]. A large number of miRNAs have been implicated in the progression of CVD. Injury caused by CVD to the cardiac muscle causes these miRNAs to be released in circulation. MiRNAs released in circulation are highly stabile, easily detectable and provide consistent results in expression levels. Due to this stability and consistency, miRNAs are receiving a lot of interest as diagnostic biomarkers for numerous chronic diseases such as diabetes, cancer, and cardiovascular disorders.

Secondly, it is well established that exercise has cardioprotective effects and interestingly, exercise has also been shown to modulate expression of circulating cardiovascular miRNAs [4]. In this review, we report an overview about the role and involvement of miRNAs in T2DM- associated heart disease and the effect of exercise intervention on miRNA regulation and expression.

## Relationship between T2DM, CVD and Exercise

The relationship between T2DM and CVD is well established. Mechanistic studies that have sought to determine the relationship between DM and CVD, have shown that there is a significant overlap in the pathological pathways of these diseases. For instance, the core defects of DM, impaired glucose tolerance and insulin resistance, can contribute to endothelial dysfunction, oxidation, inflammation and vascular remodelling, all which can accelerate the atherogenesis process [8].

### Diabetic heart disease

The cardiac illness that develops in patients with type 1 and type 2 diabetes is referred to as “Diabetic heart disease” (DHD). DHD is defined by structural, molecular, and functional alterations and is an aggregation of coronary heart disease (CHD) or coronary artery disease (CAD); heart failure (HF); cardiac autonomic neuropathy ; and/or diabetic cardiomyopathy [9].The main hallmarks of a diabetic heart are diastolic dysfunction with preserved ejection fraction. These changes are a result of pathological remodelling in the heart. Examples of these pathological alterations include left ventricle (LV) hypertrophy and increases in interstitial and perivascular fibrosis which subsequently affects cardiac output [8].

### Effect of exercise on cardiovascular function in T2DM patients

**Fig. 1:**
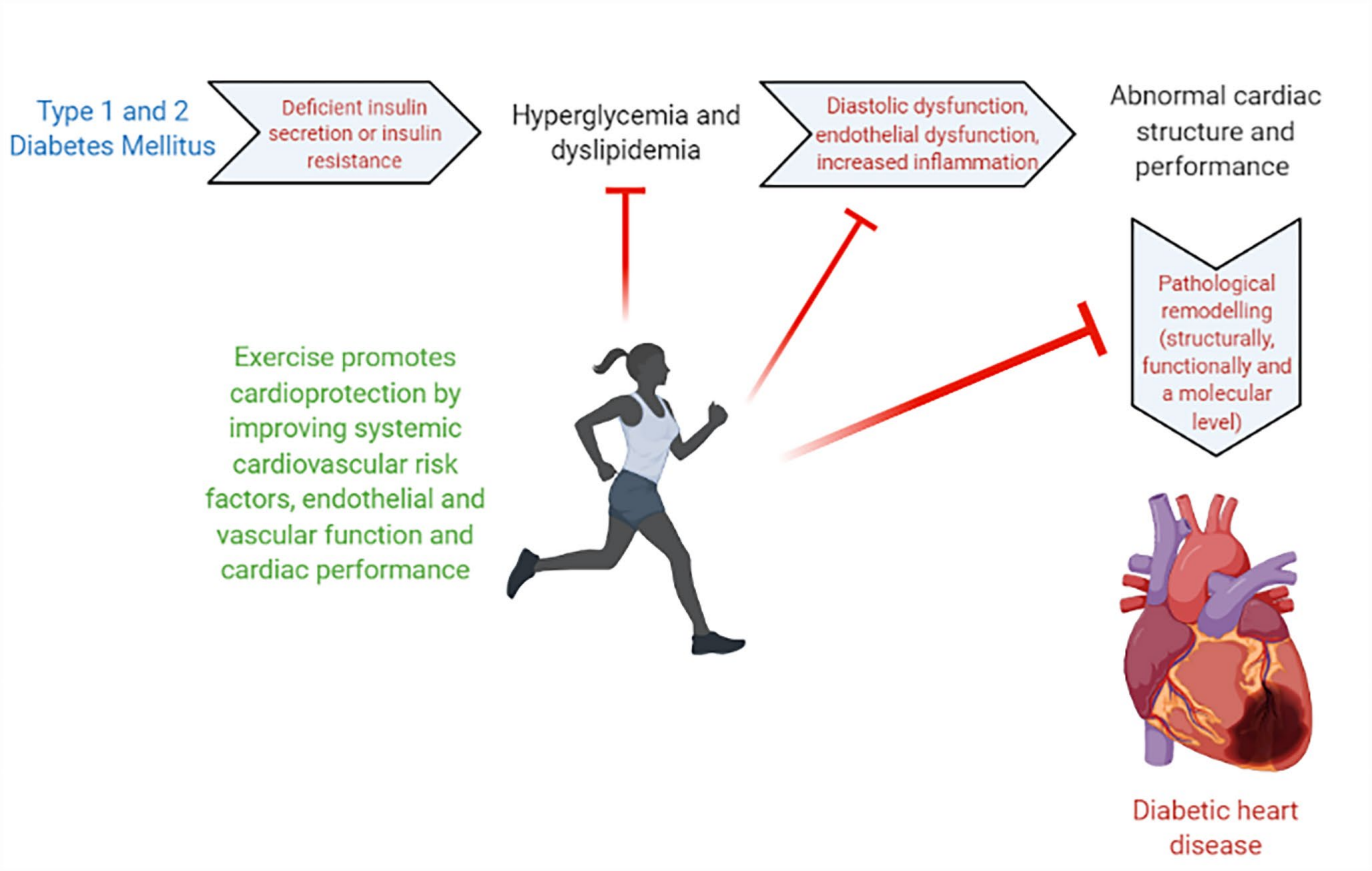
Schematic depicting the pathological events that contribute to diabetic heart disease and how exercise can prevent these effects (red arrows)

The benefits of regular physical activity on cardiovascular health are widely accepted. Regular exercise is associated with a decrease in cardiovascular mortality as well as the risk of developing CVD. Several studies have demonstrated that exercise can positively impact traditional and non-traditional CVD risk factors, causing a reduction in the risk of experiencing a cardiac event, in addition to decreasing the risk of T2DM and cancer [10] (Fig. 1). The relationship between cardiovascular risk factors and insulin sensitivity in diabetic patients is evident, and this is demonstrated through exercise research. For instance, exercise has many beneficial effects for the cardiovascular system in patients with diabetes, by providing cardio protection through the modulation of a variety of factors including systemic cardiovascular risk factors, endothelial and vascular function and cardiac performance [4]. Thus, aerobic exercise has been shown to be an effective tool in the prevention of cardiovascular dysfunction in diabetic patients and is also important for the management of blood glucose in individuals with diabetes and prediabetes [10]. The controlled glucose metabolism is a beneficial effect of performing exercise, along with anti-inflammatory effects and positive changes in body composition. Different forms of exercise when performed correctly creates distinct results such as resistance training appear to enhance insulin sensitivity primarily by gaining muscle mass whereas aerobic exercise, on the other hand, improves it by increasing the metabolic activity of the skeletal muscles [11].

As discussed above, past studies have shown that regular physical activity in diabetic patients can cause acute and chronic improvements in the body’s ability to control blood glucose levels and insulin sensitivity. Thus, as the studies discussed above suggest, identifying effective but cost-effective treatment strategies such as physical activity, for improving cardiovascular and overall health in diabetic patients, is crucial.

One study conducted by Mitranun and colleagues examined the effect of a continuous 12 week treadmill exercise program on glycaemic control and a variety of cardiovascular risk factors, in a cohort of middle-aged T2DM patients without significant diabetic, cardio or cerebrovascular diseases [12]. The findings demonstrated that both continuous and interval exercise training was effective in improving fasting blood glucose concentration, insulin resistance, aerobic fitness and lipid profiles in diabetic participants. Therefore, the authors concluded that aerobic exercise exerts beneficial effects on glycaemic control and physical fitness in patients with T2DM, which can help reduce the risk of developing vascular complications that are commonly associated with diabetes [12].

Several studies have shown that abnormal cardiac gene expression causes morphological and functional alterations in the heart, including hypertrophy, abnormal cardiac conduction, diminished contractility, and cardiomyocyte survival, as well as vascular homeostasis disruptions. Microarray analysis revealed a total of 838 genes to be differentially expressed between control and diabetic hearts, of which, 272 genes were upregulated, and 566 genes were downregulated, representing the global changes in cardiac gene expression in diabetes [13]. The identification of miRNAs as regulators of multiple cardiac genes has provided a new regulatory link at the post-transcriptional level between gene regulation in the normal and failing heart. By using an on-off switching mechanism, they could act as fine-tuners for levels of gene expression. Recently, miRNAs have been implicated as key regulators in the cardiac gene remodelling in diabetes [14]. Interestingly, studies have demonstrated the strong correlation between the structural and functional myocardial modifications observed in T2DM and changes in the expression of certain microRNAs [4]. Several recent publications have shown that T2D patients have an altered miRNA profile (Table 1). Furthermore, studies have also discovered that exercise can modulate expression of certain miRNAs (Table 2).

## MicroRNAs

MiRNAs are regulatory molecules which ‘repress’ expression of their target genes [15]. Their main mechanism of action is by partially base pairing with complementary sequences in their target mRNA (transcript). This leads to either mRNA degradation or repression of translation [16].

### MiRNA biogenesis

**Fig. 2:**
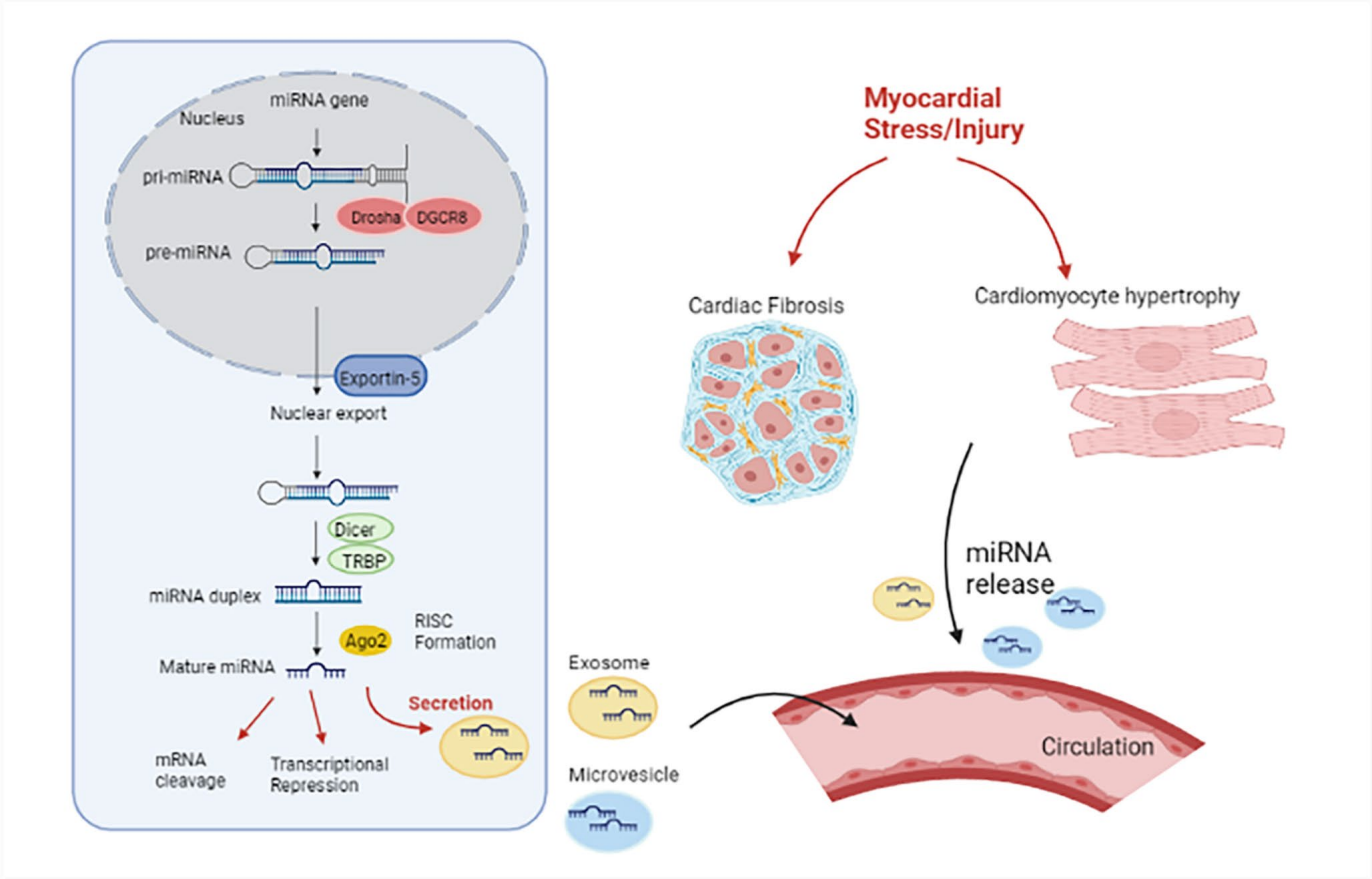
miRNA biogenesis

MiRNA biogenesis starts when the miRNA gene is transcribed by RNA polymerase II to generate a long primary transcript, termed a pri-miRNA (Fig. 2). This pri-miRNA is then processed by a complex containing an endoribonuclease called Drosha and its co-factor DiGeorge syndrome critical region 8 (DGCR8), to create a pre-miRNA, which is a 60–70 nucleotides long hairpin structure. These pre-miRNAs are then exported from the nucleus into the cytoplasm (by exportin-5). Here they undergo further cleavage by Dicer (another endonuclease) and its cofactor TAR RNA-binding protein (TRBP) [17]. This leads to the formation of an imperfect double stranded miRNA:miRNA* duplex which is 22 nucleotides long. This duplex consists of a guide (miRNA) and passenger strand (miRNA*). The guide strand is loaded onto Argonaute (AGO) proteins, creating the RNA-induced silencing complex (RISC) while the passenger strand is degraded [15]. The mature miRNA acts a guide for the RISC complex to recognise the complementary sequences located in the 3′ - untranslated region (3′ -UTR) of the target mRNA. This leads to either transcriptional repression or mRNA destabilization [16] .

miRNA genes undergo transcription by RNA polymerase II to create pri-miRNAs. These are then processed by a multiprotein complex consisting of DROSHA and DGCR8 and this leads to the generation of pre-miRNAs. The pre-miRNA is approximately 70 nucleotides long and has a short stem and a 3’ overhang. After being transported into the cytoplasm by Exportin-5, they are cleaved by DICER in complex with TRBP. This results in the formation of a mature duplex consisting of a guide and passenger strand. The guide strand is loaded together with Argonaute (Ago2) proteins into the RISC complex while the passenger strand is degraded. The mature miRNA acts as a guide for the RISC complex to recognise the complementary sequences located in the 3′ -UTR of the target mRNA (and directing its repression). Furthermore, upon stimulation or injury, miRNAs can also be secreted by cells through microparticles (exosomes, microvesicles, and apoptotic bodies) which are then released into the extracellular space or into circulation. For example, myocardial stress can induce pathological changes such as cardiac fibrosis and hypertrophy and lead to subsequent miRNA release from damaged cells.

### Mechanisms of circulating miRNAs secretion

As miRNAs are involved in regulating gene expression, they were initially thought to be only intracellular. However, subsequent studies discovered that miRNAs could exist in ‘a cell free’ circulating form in the bloodstream (plasma or serum) or other biological fluids [18].

The exact mechanisms by which circulating miRNAs are released into are unclear. However, previous studies have described that miRNA can be released into circulation after stimulation or injury (Fig. 2). They can be released in several packaged forms such as extracellular vesicles (including exosomes, micro vesicles, high-density lipoprotein, and apoptotic bodies) [19]. Since these miRNAs are encapsulated in microparticles, this prevents them from getting degraded (by RNA-degrading enzymes) in serum or plasma and they can remain stable in circulation. In fact, microparticles are a form of cell-to-cell communication. MiRNAs released from one cell type can be effectively taken up by another cell and influence the recipient cell. An example would be apoptotic bodies, containing miR-126 that can be released from the endothelium and delivered to into atherosclerotic lesions which can send alarm signals to recipient vascular cells, causing the recruitment of progenitor cells and reduce atherosclerosis [20].

The two main mechanisms of miRNA release from cells are active secretion or passive release, depending on the stimulus [19]. Cardiac stress, including cardiac ischemia and volume/pressure overload can initiate miRNA mobilization via exosomes (active secretion) or from cells (e.g., myofibers) that underwent necrosis (passive secretion). These exosomes fuse with the cell membrane of their target (recipient) cell, delivering their genetic contents into the cell [21].

MiRNAs have been designated as the micro regulators of gene expression. They play a crucial role in expression and regulation of signalling pathways for proper balanced functioning of the cell. As miRNAs can act as regulators of many cardiac genes, their dysregulation would be linked to pathogenesis in the diseased heart. MiRNAs have recently been discovered as a critical component in cardiac gene remodelling in diabetic hearts [14]. MiRNAs have been implicated in the pathophysiology of diabetes and a variety of cardiovascular problems, including endothelial dysfunction, angiogenesis, hypertrophy, arrhythmia, HF, and myocardial fibrosis [14] by regulating the expression of multiple genes (See Table 1). As these processes involve the dysregulation of multiple genes, it is reasonable to hypothesize that miRNAs could be implicated in the pathogenesis of DHD.

### MiRNAs and DM

Several studies have investigated the circulating miRNA profiles in diabetic patients, and these are described in Table 1. These miRNAs are involved in different processes such as glucose metabolism (e.g., miR-375), inflammation, endothelial dysfunction and angiogenesis [22]. As seen in Table 1, there is a limited number of studies which have examined miRNA expression exclusively in diabetic patients who have CHD or other CV complications. Amr et al., in their study discovered that T2DM patients with CAD had lower levels of plasma miR-126 (lower by 13.1-fold) than T2DM patients without CAD (lower by 2.8-fold) when compared to healthy subjects [23]. Several studies have discovered that T2DM is associated with reduced expression of miR-126 (see Table 1). The role and function of this miRNA will be discussed in the next section.

**Table 1:** Summary of studies investigating expression of miRNAs in T2DM patients. As the list of miRNAs studied is extensive, only those which are involved in DHD pathogenesis will be discussed in the text

Study	Study Participants	Source	Method of Analysis	Expression of miRs in T2D	Findings
Kong et al. [24]	8 Newly diagnosed T2DM patients and 19 T2DM-susceptible individuals	Serum	qRT-PCR	↑ miR-9 ↑ miR-29a ↑ miR-30d ↑ miR-34a ↑ miR-124a ↑ miR-146a ↑ miR-375	Expression of miRNAs were significantly elevated (p < 0.05) in newly diagnosed T2DM patients compared to T2DM-susceptible individuals
Zhang et al. [25]	30 T2DM patients, 30 T2DM-susceptible and 30 normal individuals	Plasma	qRT-PCR	↓ miR-126	Expression of miR-126 was significantly lower in susceptible and T2DM groups compared with the normal group (p < 0.01)
Liu et al. [26]	160 patients with newly diagnosed T2DM, 82 subjects with impaired glucose tolerance (IGT), 75 subjects with impaired fasting glucose (IFG) and 138 healthy individuals	Serum	qRT-PCR	↓ miR-126	MiR-126 concentration was significantly lower in IGT/IFG and T2DM patients than in healthy controls (p < 0.001)
Al-Muhtaresh et al. [27]	30 T2DM patients ,30 prediabetes patients and 30 healthy controls	Whole blood	qRT-PCR	↑ miR-375 ↑ miR-9	Both were expressed at higher levels in prediabetes patients and progressively more enriched in T2DM patients compared to controls (p < 0.05)
Zampetaki et al.[28]	80 T2DM patients and 80 matched controls	Plasma	microRNA profiling and qRT-PCR	↑ miR-28-3p ↓ miR-24 ↓ miR-21 ↓ miR-20b ↓ miR-15a ↓ miR-126 ↓ miR-191 ↓ miR-197 ↓ miR-223 ↓ miR-320 ↓ miR-486 ↓ miR-150 ↓ miR-29b	The differences in miRNA levels between patients and controls were significant (p < 0.05) for the following miRNAs: miR-24, miR-21, miR-15a, miR-126, miR-191, miR-197 and miR-223
Al-Kafaji et al. [29]	45 T2DM patients without chronic complications, 45 T2DM patients with CAD and 45 healthy individuals	Whole blood	qRT-PCR	↓ miR-126	Expression of miR-126 was significantly lower in T2DM patients (by 4.2-fold) and T2DM patients with CAD (by 7.7-fold) than in healthy subjects (p < 0.05)
Luo et al. [30]	36 T2DM patients with CHD, 43 diabetic individuals with no complications, 48 pre-DM subjects and 46 healthy subjects	Leukocyte-depleted platelets	qRT-PCR	↓ miR-103b	Expression of miR-103b was significantly decreased in patients with pre-DM, T2DM and CHD, and T2DM individuals with no complications when compared to the control (p < 0.05)
Deng et al. [31]	28 T2DM patients with CHD, 35 CHD patients and 31 control subjects	Peripheral blood	qRT-PCR	↓ miR-24	Levels of miR-24 were decreased in both T2DM patients with CHD and patients with CHD alone when compared to control subjects (p < 0.05)
Amr et al. [23]	100 T2DM patients (54 without CAD, 46 with CAD) and 20 healthy subjects	Plasma	qRT-PCR	↓ miR-126 ↑ miR-210	A significant decrease of miR-126 and increase of miR-210 observed in diabetic patients with and without CAD compared to controls (p < 0.05)
Al-Muhtaresh et al. [32]	30 T2DM patients, 30 T2DM patients with CAD and 30 healthy controls	Whole blood	qRT-PCR	↑ miR-1 ↑ miR-133	Levels of miR-1 were increased in T2DM patients (p = 0.005) and in T2DM patients with CAD when compared to controls (p < 0.001). Levels of miR-133 were higher in both disease groups when compared to controls (p < 0.001)
Baldeón et al. [33]	56 Ecuadorian T2DM patients and 40 healthy controls	Serum	qRT-PCR	↓miR-146a	Levels of miR-146a were significantly reduced in T2DM patients as compared to the non-diabetic controls (p = 0.04)
Zeinali et al. [34]	30 T2DM patients, 30 prediabetic patients and 30 healthy controls	Whole Blood	qRT-PCR	↑ miR-122 ↓ miR-126-3p ↓ miR-146a	Levels of miR-122 were significantly higher and expression of miR-126-3p and miR-146a were significantly lower in T2DM and prediabetic group when compared to the controls (p < 0.001)
Ahmed et al. [35]	50 T2DM patients with cardiomyopathy (had DM > 10 years) and 50 healthy controls	**Buffy coat and Plasma**	qRT-PCR	↑ miR-17 ↓ miR-24 ↓ miR-150 ↓ miR-199a ↓ miR-214 ↓ miR-320a	Expression of miR-17 was increased while levels of remaining miRs were decreased in T2DM patients with cardiomyopathy vs. controls (p between 0.0003–0.0009)
Al-Hayali et al. [36]	45 T2DM patients, 45 T2DM patients with CAD, 45 T2DM patients with acute HF and 45 healthy controls	Serum	qRT-PCR	↓ miR-1 ↑ miR-21	Levels of miR-1 were significantly lower and levels of miR-21 were significantly higher in all 3 T2DM groups when compared to controls (p < 0.001)

### Pathophysiological role of cardiovascular miRNAs in DHD

Cardiovascular miRNAs (CV miRNAs) are a subgroup of miRNAs that are either highly or selectively expressed in vascular and cardiac cells. Throughout the past 10 years, miRNAs have been discovered to modulate the cardiovascular system and are emerging as biomarkers for CVD states such as cardiac hypertrophy and acute HF [37]. In this section of the review, we will be focusing on specific CV miRNAs which could be involved in the pathogenesis of DHD and could be used as potential biomarkers.

Cardiac Hypertrophy (CH) is a major compensatory mechanism of the heart in response to different pathophysiological signals such as myocardial stress because of injury, etc. [38]. At first, CH is a compensatory mechanism which is employed by the heart to reduce stress on its walls and improve cardiac output. Unfortunately, if CH is extended, it can lead to contractile dysfunction, heart decomposition and lastly HF [39]. MiRNAs such as miR-133 and miR-1, which belong to the same transcriptional unit, have been discovered to play a pathogenic role in CH [40]. Both miRNAs are only expressed in cardiac and skeletal muscles [37]. Sayed et al. [41] conducted a study to analyse miRNAs whose expression was altered in CH. Analysis was carried out from the hearts of mice 1, 7, and 14 days after they had undergone transverse aortic constriction or a sham operation. The results showed that expression of miR-1 was reduced after onset of pressure overload on the heart at the start and progression of CH. Furthermore, Care et colleagues [42] found that expression of both miR-1 and miR-133 were downregulated in both mouse and human models of CH suggesting that they could serve as markers of heart disease. Contrastingly, researchers have found that the expression of these miRNAs is in fact upregulated in other cardiovascular conditions such as AMI, CAD [6, 43].

MiR-208 is a ‘cardiac-enriched’ miRNA. MiR-208 has 2 subfamilies, miR-208a and miR-208b. MiR-208a is encoded within an intron 29 of the Myh6 encoding alpha myosin heavy chain (αMHC). It is only expressed in cardiac muscle. MiR-208b is encoded within an intron 31 of Myh7 gene encoding beta MHC (βMHC) and it is expressed in both skeletal and cardiac muscles [37]. The contractility of the heart is dependent on expression of these two *MHC* genes (α and β). The heart responds to various stress signals by hypertrophy (along with fibrosis) and a subsequent reduction in contractility (due to downregulation of αMHC and upregulation of βMHC) [44]. Dilated cardiomyopathy (DCM) is the most common type of cardiomyopathy (disease of the myocardium). Satoh and colleagues [45] conducted a study to investigate expression of miR-208 and miR-208b in patients with DCM. Levels of these miRNAs were analysed from endomyocardial tissue biopsies from DCM patients or individuals without LV dysfunction. The findings were that expression of miR-208 and miR-208b were elevated in DCM patients when compared to the controls. Additionally, DCM patients had lower mRNA levels of α-MHC and higher levels of β-MHC. Finally, after follow-up, it was found that higher levels of miR-208 was associated with worse clinical outcomes, suggesting miR-208 could be a strong prognostic marker in patients.

MiR-499 is a miRNA that is ‘muscle-specific’ and elevated levels of this miRNA has been associated with myocardial damage in CVD [46]. Corsten et al. [46] analysed miRNA expression in plasma samples from patients with varied extents of heart damage, including patients who had acute myocardial infarction (AMI), viral myocarditis, diastolic dysfunction, or acute HF. They found that AMI patients had significantly increased levels of miR-208b (by 1600-fold) and miR-499 (by 100-fold) when compared to controls. Furthermore, Shieh et at discovered that increased levels of miR-499 ‘blunts’ cardiac stress response and alters the expression of cardiac genes [47].These studies indicate that miR-499 may also play a role in DHD pathophysiology.

MiR-132 is not ‘cardiac-specific’ but has been implicated in cardiac pathophysiology. A study conducted by Ucar et al. [48] reported that hypertrophic stimuli increased expression of miR-212 and miR-132 in cardiomyocytes. Additionally, the authors in this study found that transgenic (TG) mice which were overexpressed with miR-212 and 132, had significantly enlarged hearts and a reduced life expectancy. Hearts from the TG mice also had elevated expression of atrial and brain natriuretic peptides (ANP and BNP respectively) which are cardiac stress markers. These results suggest that miR-132 plays a pivotal role in cardiac failure.

MiR-126 is expressed exclusively in endothelial cells. This miRNA is reported to have a ‘cardioprotective’ and ‘vasculoprotective’ role. Zampetaki et al., [28] in their study, also investigated if high glucose affects miR-126 release from endothelial cells (ECs) by analysing miR-126 levels in apoptotic bodies released from ECs cultured in either normal or high glucose conditions. Hyperglycaemic conditions significantly decreased miR-126 content in endothelial apoptotic bodies, but cellular miRNA concentration was unchanged. To summarise, the main finding from this study was that T2DM is associated with loss of miR-126. As shown in Table 1, several studies have reported similar findings. This is significant as this miRNA is involved in the regulation of vascular integrity and vascular endothelial growth factor (VEGF) signalling [49]. Shedding of miR-126 from ECs has been shown to modulate VEGF responsiveness and mediate vascular protection. Thus, low levels of plasma miR-126 (observed in DM) suggests that less is delivered to other cells such as monocytes which may give rise to VEGF resistance and endothelial dysfunction [28].

Interestingly, numerous studies have found that exercise can alter the expression of these circulating cardiovascular miRNAs and these are listed in Table 2.

## Exercise and miRNA expression

Regular physical activity is an important part of leading a healthy lifestyle, as it can help to prevent and reduce the risk of diseases such as metabolic and aging-related diseases, as well as cancer, and has an impact on mitochondrial metabolism, as well as cognitive, cardiovascular, and immune functions [50]. Furthermore, due to exercise-induced cardiovascular advantages, certain training programs have become non-pharmacological treatments for reducing cardiovascular morbidity and mortality [51]. Exercise training can affect many signalling pathways, which can affect a variety of exercise-related features like energy metabolism, angiogenesis, and inflammation. This can have a significant benefit to health and reduce the risk of chronic illness.

MiRNAs have recently been discovered in bodily fluids such serum, plasma, urine, saliva, and cerebrospinal fluid. As a new form of intercellular communication, circulating miRNAs are persistent, easily detectable, and may influence gene expression in target cells and tissues [52]. Circulating miRNAs are now being described as possible clinical biomarkers for particular illnesses and the administration of pharmacological drugs, according to growing data [53]. Exercise-induced alterations in circulating miRNAs have been frequently observed in both healthy people and patients, indicating that miRNAs may play a role in physiological adaptations to exercise. The profiles of circulating miRNAs change depending on the kind, duration, and intensity of exercise [53].

### Importance of measuring miRNAs in exercise interventions

As exercise has been shown to be an effective tool in improving cardiovascular health and fitness in diabetic patients, it’s vital that researchers have a method that can accurately and physiologically track the progression of cardiovascular and health improvements during an exercise intervention. One possible way to do so is to measure the regulation and changes in circulating levels of miRNA, before, during and after an exercise program. As previously mentioned, past research has demonstrated that circulating miRNA levels in human plasma can be used as biomarkers for individual responses to different exercise modalities, intensities and durations, and reflect physiological and pathological states [54]. In addition, measuring the plasma levels of organ specific circulating miRNAs may provide information regarding specific organ function and structure [4]. This is essential because it is important to identify the role of miRNAs in the development of a disease, from a physiological and mechanistic perspective, in order to better understand how exercise can mediate its effects and the efficacy of the treatment.

### Exercise and miRNA levels in T2DM patients

The main objective for this review is to examine the beneficial cardiovascular effects of exercise on diabetic patients, and to determine whether these health improvements are reflective of changes in physiologically relevant miRNAs. Several studies have demonstrated that circulating miRNA levels may serve as a potential biomarker for the monitoring and improvement in cardiovascular function in response to an exercise intervention (see Table 2). For instance, in an animal study conducted by Lew et al., the authors sought to investigate the effectiveness and mechanisms of exercise-induced coronary and cardiac protection, in a cohort of diabetic and non-diabetic mice with or without cardiac dysfunction, as well as explore the potential role of miRNAs as novel biomarkers for measuring the progression of DHD [4]. Of particular interest in this study was miR-126, a proangiogenic miRNA that is usually downregulated in the early stages of T2DM and linked to impaired coronary function [4]. After 8 weeks of moderate and high intensity physical activity, the authors demonstrated that the exercise intervention prevented DM-induced onset of systolic and diastolic dysfunction and cardiac structural remodelling in mice subjects, as well as normalize and even increase the expression of miRNAs, compared to their non-exercise counterparts [4]. Subsequently, further analysis demonstrated that the beneficial effects of exercise exhibited in this study, are partially mediated by the changes in expression of miR-126, as there was a significant positive correlation between miR-126 levels and coronary arteriole density and capillary density in subjects [4]. This study provides evidence that miRNAs can serve as potential biomarkers in diabetic individuals and those levels of cardiac-enriched miRNAs, which can be altered via exercise, have an important mechanistic role in the development of DHD. Similarly, in a study conducted in a cohort of young, healthy male participants, Aoi and authors [54] investigated the effect of acute and chronic aerobic exercise on the levels of muscle-enriched circulating miRNAs. Following 4 weeks of the intervention, both acute and chronic exercise caused a significant reduction in the levels of muscle-enriched miR-486. These findings demonstrate that circulating levels of miR-486 may mediate various metabolic changes and adaptation during exercise training. Therefore, as changes in the level of circulating miRNAs in response to various exercise interventions reflect improvements in health, this provides support that miRNAs may serve as potential biomarkers for disease diagnosis and progression, particularly in diabetic patients.

The studies discussed above were focused on the effect of aerobic exercise on miRNA expression. However, resistance exercise has also been found to alter levels of circulating miRNAs. Mueller et al. [55] discovered that regardless of the strength regimen employed, there is a decrease in miR-1 expression, an increase in IGF-1 (insulin-like growth factor-1) expression, and a rise in skeletal muscle mass. These findings were achieved in older participants who completed a 12-week strength-training program that included both traditional resistance exercise and eccentric ergometer training [56]. Not only were miR-136-5p and miR-376a-3p differently expressed at baseline, but they were also variably regulated by acute and chronic resistance exercise in healthy young men.

In another study, resistance exercise training was carried out for 16 weeks in middle-aged T2DM obese Polynesian individuals, which resulted in unique DNA methylation, gene, and miRNA expression changes when compared to aerobic exercise [57]. Resistance training caused changes in miR-1207-5p and miR-195, with miR-195 showing upregulation. Additionally, in T2DM individuals, an acute resistance intervention administered as a circuit training led to a greater rise in total circulating levels of miR-146a than in non-diabetic participants [58]. In acute aerobic intervention, however, there were no changes in circulating levels of miR-146a, miR-126, or miRNA-155.

**Table 2:** Characteristics of microRNA investigated in exercise studies

miRNAs	Target organ/ system	Phenotype influenced	Exercise modality	Training Protocol and sample collection	Subjects	Results (p < 0.05)	Reference
miR-1	Skeletal muscle	Modulator of muscle cell function (muscle atrophy, myogenesis and muscle cell proliferation)	Strength training	Control and strength training group	Prostate cancer patients	The expression fold-change of miR-1, miR-29 and miR-133 in the experimental group were significantly increased, in response to 16 weeks of strength training program and androgen-deprivation therapy	[59]
miR-126	Heart	Pro - angiogenesis and vascular homeostasis	Aerobic	Moderate (65–70% VO_2_ max) and high intensity (85–90% VO_2_ max) exercise on a treadmill 5 days/week for 8 weeks (10 bouts of 5 min).	Mice model Diabetic (DM) and non-DM, with and without underlying cardiac dysfunction	Upregulating miR-126 restored the protective capacity of moderate-intensity exercise in mice with advanced DM Downregulated miR-126 was associated with DM and coronary artery disease, while silencing miR-126 signalling abolished the exercise protection in mice with early DM	[4]
miR-132	Immune system	Regulatory inflammatory function: immune cell function and regulation of circulating leukocytes and toll-like receptors in monocytes	Aerobic	20 min (two 10-minute bouts) of cycle ergometry	Young, healthy men	Expression of miR-132 was up-regulated following acute exercise	[60]
miR-133	Heart	Regulator of cardiac hypertrophy (anti-hypertrophic)	Aerobic	Swimming 2 training groups with slightly different exercise timelines (moderate and high-volume)	Mice model Control (sedentary) and exercise group	miR-133a and 133b were down-regulated in both swimming groups, compared to sedentary animals	[61]
miR-208 (a and b)	Heart	Cardiac hypertrophy	Aerobic	Swimming (high-volume, low intensity) Goal of 2 protocols: protocol 1 (T1) = promotion of cardiovascular adaptations and protocol 2 (T2) = induce greater cardiac hypertrophy and aerobic high performance	Mice model Control (sedentary) and exercise group	High-volume swim training decreased the expression of miR-208a and 208b There was a significant decrease in miR-208b expression between T1 and T2 groups, compared to the control (sedentary) group	[62]
miR-486	Skeletal muscle	Modulator of muscle cell function and regulation of glucose metabolism	Aerobic	Cycle training 3 days/week for 4 weeks Acute bout (60 min) of steady state cycling at 70%	Healthy, young men	miR-486 was decreased by both acute and chronic exercise Negative correlation between change ratio of miR-486 (during acute exercise) and VO_2_ max	[54]
miR-499	Skeletal muscle	Regulator of muscle fitness and endurance	Aerobic	Marathon (42 km run)	Healthy, male marathon runners	Levels of miR-499 significant increased post-exercise	[63]

## MiRNAs as biomarkers for CV damage in T2DM

As outlined above, circulating miRNAs can serve as markers of (heart) muscle damage. Biomarkers are biological indicators that may be objectively tested and utilized to signify a physiological, pathological, or pharmacological condition [53]. Furthermore, they can give data on both normal molecular physiology and disease activity and progression. Pharmacologists also employ them to obtain insight into the molecular action of medicines, as well as their efficacy and safety.

Several clinical indicators have been linked to cardiovascular events in recent years. C-reactive protein (CRP), cardiac troponins I and T (cTnI and cTnT), B-type natriuretic peptides (BNP and NT-proBNP), and D-dimer are some of these biomarkers [64]. CRP is a pattern recognition molecule that is increased in inflammatory diseases like atherosclerosis. CRP levels are closely linked to future cardiovascular risks, and increased CRP levels indicate cardiovascular morbidity [65]. The cardiac troponins cTnI and cTnT are important biomarkers for detecting acute MI and risk stratification in acute coronary syndrome [66]. BNP and NT-proBNP (BNP and NT-proBNP) are B-type natriuretic peptides that are utilized as biomarkers to detect heart failure in both acute and chronic conditions. Thrombosis, cardiovascular mortality, acute aortic dissection, and ischemic heart disease are all biomarkers for D-dimer [66]. Although these indicators are often employed in clinical practice and have assisted doctors in saving lives, they only identify cardiovascular events after they have happened (late-stage biomarkers). For this reason, it has become important to identify biomarkers that can detect early-stage CVD and can also be used to monitor changes the predict disease progression or improvement in CV function in order to minimize morbidity and death from cardiovascular events while also improving prognosis.

Circulating miRNAs have many features which make them a suitable candidate as a biomarker. For example, miRNA analysis in the laboratory is quick and cost-effective. MiRNAs are stable in different body fluids such as plasma and can be detected rapidly and accurately [67]. Quantification of circulating miRNAs can be performed using real-time quantitative reverse transcriptase-polymerase chain reaction (qRT-PCR), using highly specific and sensitive assays [7]. Since circulating miRNAs can readily detected by PCR, this is one advantage they have over the protein-based biomarkers discussed above (where low amounts can affect detection) [67]. Furthermore, some studies suggest that the altered expression of extracellular miRNAs may correlate with the extent of T2DM (and its associated cardiovascular complications) [68]. For example, Kong and colleagues discovered that the levels of a panel of miRNAs were significantly (p < 0.05) increased in T2DM patients compared to those that were T2DM-susceptible [24]. As shown in Table 1, Zeinali et al. [34] discovered that expression of circulating miR-126-3p was significantly lower in T2DM patients (fold change = 0.09 ± 0.09) and pre-diabetic patients (fold change = 0.29 ± 0.12) when compared to healthy individuals (fold change = 1.07 ± 0.40) (p < 0.001). Importantly, levels of circulating miR-126-3p were significantly decreased in T2DM patients versus pre-diabetic patients (p < 0.001). This indicates that miRNAs can possibly aid early detection (before onset of clinical symptoms) and are sensitive to changes in the disease. This will be useful for monitoring disease progression and treatment response in diabetic patients.

Additionally, Al-Muhtaresh and colleagues [32] identified that levels of circulating miR-1 and miR-133 were significantly elevated in T2DM patients without CAD and progressively increased in T2DM patients with CAD when compared to healthy individuals. The levels of miR-1 were 2.1-fold higher and levels of miR-133 were 1.5-fold higher in T2D patients with CAD compared to those with T2DM alone (p = 0.002 and p = 0.003 respectively). Contrastingly, Al-Hayali et al. [36] discovered that miR-1 levels were significantly lower (by 0.22-fold) in T2DM patients with HF and lower (by 0.22-fold) in T2DM patients with CAD than in patients with T2DM alone (p < 0.001). Even though these studies yield contradicting results, it highlights the need for future research to fully elucidate the role of miR-1 and miR-133 (and other cardiovascular miRNAs) and evaluate their ‘biomarker potential’ in DHD.

**Fig. 3:**
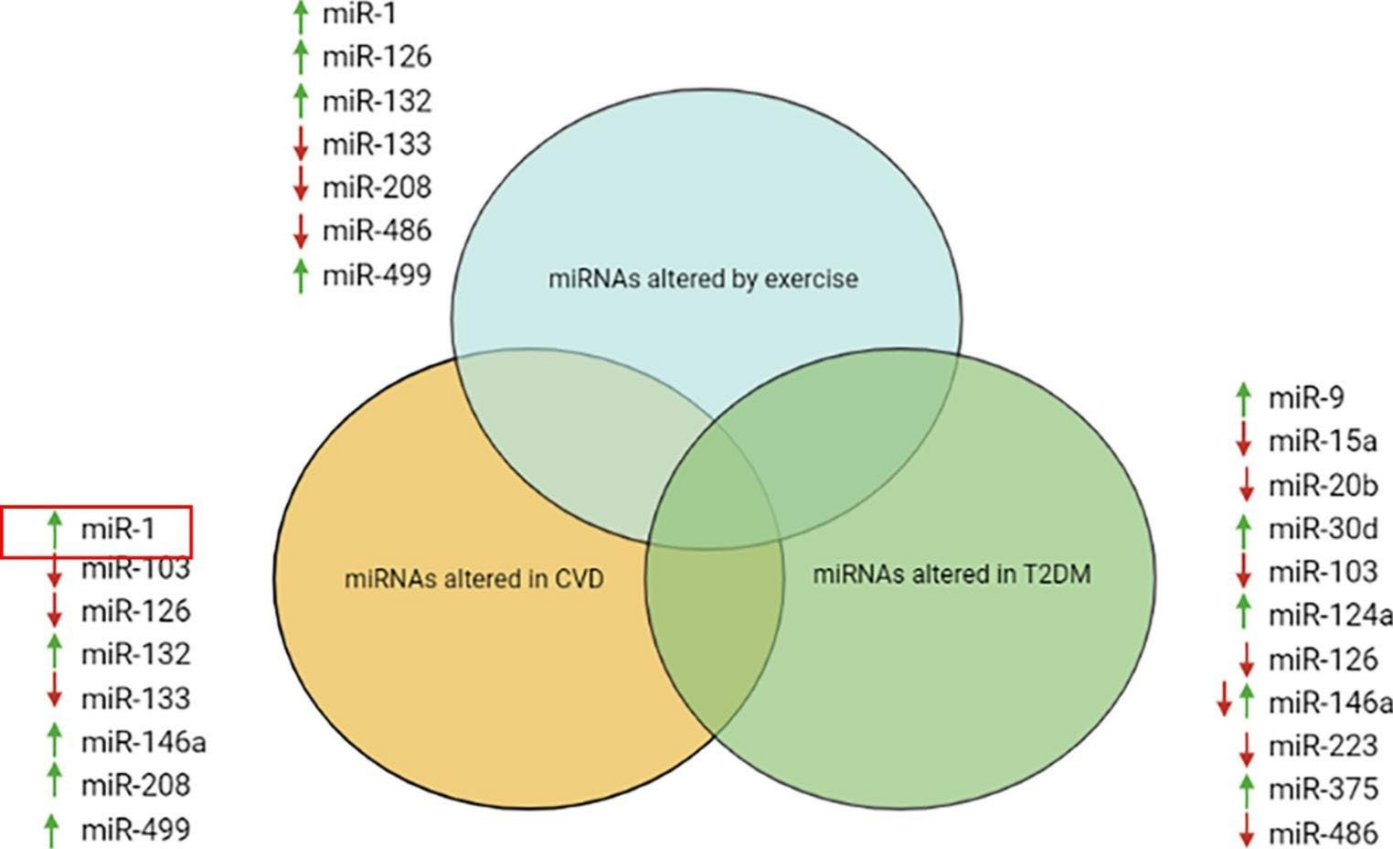
Venn diagram showing miRNAs whose expression was found to be altered by exercise, CVD and T2DM in previous studies. ↑ = up regulated, ↓= down regulated and ↑↓ = expression of the miRNA found to be up and down regulated in different studies

Despite their promise, miRNAs still have not entered the clinical setting, mainly because of a lack of large cohort studies [7]. As discussed previously in this review, there is extensive literature linking DHD (and CVD) development with altered miRNA expression. Unfortunately, T2DM patients with CVD complications are often excluded in miRNA studies. Secondly, there are a limited number of studies investigating effect of exercise on miRNA expression exclusively in diabetic patients with heart disease. As seen in Fig. 3, there is an overlap in the miRNAs that are altered by exercise, CVD and T2DM. Key examples include miR-103, miR-132, miR-146a and miR-499. The most important of these findings is miR-126 whose expression is found to be decreased in both disease states but increased by exercise, indicating that it may be a useful biomarker of DHD. Certain miRNAs including miR-146a and miR-499 (elevated in CVD but also by exercise) have found to have contrasting results so further study is required in this area. Future research should focus on uncovering a distinct ‘miRNA profile’ for DHD patients. The next step would be to observe the effect of exercise trials on the expression of this miRNA profile in DHD patients.

## Conclusion

This review article provides a detailed and extensive discussion on miRNA signatures that are affected by T2DM and DHD. The review also aims to identify a subset of miRNAs that are also affected by different types of exercise. This approach helps us to identify those miRNAs whose expression and function can be altered by regular bouts of exercise. These miRNAs can be correlated to different physiologic parameters improved following regular exercise. These miRNAs can therefore serve as a tool to monitor the cardio-protective, anti-inflammatory and metabolic effects of exercise in people suffering from T2DM. Future research should focus on molecular mechanisms involved in the regulation of these miRNAs in T2DM and how they can be altered by appropriate lifestyle factors including exercise. Research should also be focused on circulating miRNA signatures associated with physiologic parameters improved following regular exercise. The data obtained will aid in the development of miRNA signatures that may be used to predict diabetes induced onset of cardiovascular changes and progression of DHD in patients with T2DM. Furthermore, collecting data samples, standardizing miRNA detection technologies, and tracking and confirming disease-state correlations will increase their prognostic and diagnostic utility for designing therapeutic intervention strategies.

## Electronic supplementary material

Below is the link to the electronic supplementary material.

Supplementary Material 1
