# Supplementary material for: MicroRNAs as biomarkers for monitoring cardiovascular changes in Type II Diabetes Mellitus (T2DM) and exercise
Source: J Diabetes Metab Disord. 2022 Jul 7;21(2):1819–32. doi: 10.1007/s40200-022-01066-4 (PMC9261151; doi:10.1007/s40200-022-01066-4)
Supplement: Supplementary file 1 — Supplementary Material 1 [file 40200_2022_1066_MOESM1_ESM.pdf]

NUI Galway
OÉ Gaillimh

Date: 06-01-2022

Discipline of Physiology,
School of Medicine, NUI Galway

Diabetes and Metabolism
Editorial Office,

Dear Editor
We would like to thank the editors of Diabetes and Metabolism for giving us the opportunity to submit our manuscript titled: **"MicroRNAs as biomarkers for monitoring Cardiovascular changes in T2DM and Exercise",** to be considered for publication in your journal.

We thank you in advance for your time and effort in handling this manuscript and hope that you will find that the manuscript meets the merits of publication in your journal.

Please feel free to contact me if you have any questions.

Yours Sincerely:

Ananya Gupta

Ananya Gupta (Ph.D)

Asst Professor
Discipline of Physiology
Director of MSc in Exercise physiology
Tel: 353-91492358
Email: ananya.gupta@nuigalway.ie
